# Medical and Physical Intelligence

**Published:** 1824-05

**Authors:** 


### MEDICAL AND PHYSICAL INTELLIGENCE.

### PATHOLOGY.

1. *Perforation of the Stomach, recognized during Life.*—J. B. BENEUX, aged fifty-seven, was admitted at La Charité, in December 1823, having at the pit of the stomach an indolent indistinct tumor, which appeared to have its site in the left lobe of the liver. According to the account of the patient, it had been present about twelve months, and had succeeded to a violent blow on the part. No bad effect had been produced on the health of the subject, whose appetite was good, and who had neither fever nor vomiting: he was in moderate condition, and without any appearance of emaciation, while the expression of the countenance, and general aspect, conveyed no idea of any chronic disease. He remained in this state nearly a month, and M. LAENNEC began to doubt the existence of the tumor, which at times was very indistinct, and suspected that M. Beneux only wished to take up his quarters in the hospital for the winter. In the beginning of January, however, he began to experience nausea and vomiting: these symptoms at first came on only in the morning, and the egesta consisted of stringy mucus, without smell. Soon afterwards, the aliments were vomited a short time after they had been received into the stomach; and from this period the patient began to lose flesh, the skin at the same time becoming sallow. The tumor remained without perceptible change, except that some pain was now excited by strong pressure. M. Laennec now suspected schirrus of the stomach. On the 15th of January, the matters vomited began to assume a brown appearance, and soon became exactly like coffee-grounds. On the 19th, the patient was seized all at once with acute pain in the abdomen, which came on immediately after swallowing some soup. At ten in the morning, when M. Laennec saw him, the abdomen was extremely painful on pressure, and sounding externally *(resonnant exterieurement)* on very gentle percussion; the features pinched; the pulse contracted, very small, and very weak. Judging from these symptoms, M. Laennec made the following remark in the report: "Ventriculus hodie ruptus est in loco schirri, et jam peritonitis adest et tympanitis abdominalis." In explaining afterwards the reasons for this diagnosis, he founded it principally upon the sudden appearance of peritoneal tympanitis, which he recognized by a sensation analogous to that which a sheet of fine parchment, placed within the cavity of the abdomen, might have produced, and which he experienced in pressing the anterior part of the belly, or striking it very gently. As to the rest, this sensation was fugacious, and scarcely any of the pupils could discern it distinctly. Several leeches were applied to the left side, and the patient put on rigid diet. Some relief was given by these means, which were repeated next day; but the patient gradually sunk, and died on the evening of the 24th, having become inconceivably emaciated during the last few days. The appetite, however, had returned, and some soup, which was allowed him, neither increased the pain nor vomiting.

*Examination of the Body.*—The abdomen, on being struck, emitted the same lymphatic sound which had been perceptible during life. On cutting through the integuments, a considerable quantity of air was evacuated, which had little smell, and evidently came from the cavity of the peritoneum. This cavity contained, besides, about two pints of fluid, having a yellowish-brown colour, with numerous irregular flocculi. The liver was considerably diseased; but the principal interest was, of course, attached to the state of the stomach. On its anterior surface, it presented the appearance of furrows, converging towards the centre of the lesser curvature; being raised with caution an oval-shaped ulceration was discovered on the posterior surface, and in the centre of this was a small perforation, through which the contents of the stomach had escaped. Being cut open along the great curvature, it presented internally a much more extensive ulceration, round which the coats of the viscus were considerably thickened and indurated, with a greyish semi-transparent matter connected with them. These appearances diminished in proportion as the distance from the ulceration increased, and finally vanished. The pylorus was not diseased. The pancreas, which was intimately connected with the stomach, was converted into a firm, white, medullary-looking substance; its rounded end alone being in a healthy state.—(*Revue Medicale*, Mars.)

---

### THERAPEUTICS.

2. *Convulsions, caused by Worms, cured by injecting Tartar Emetic into the Veins.*—Dr. MEPLAIN, of Donjon, in the department of the Allier, is the author of this interesting case. This gentleman was called upon to visit a girl, twenty-two years of age, of a lymphatic temperament, short and fat, who had suffered, from her infancy, from attacks of worms, but was in other respects healthy. For upwards of a fortnight previously, her appetite had been voracious; yet, notwithstanding she was always hungry, the sight and smell of food disgusted her, and she really ate but little; her nights were disturbed by frightful dreams; in the morning she complained that her mouth was full of a mawkish saliva; she had frequent inclination to vomit. At the termination of a journey, in which she had suffered a good deal from cold and damp, she was seized in the evening with a violent attack of fever, with intense head-ache and eructations, wandering pains in the limbs, and great restlessness. The night passed badly, with violent cramps in the legs, low delirium, and grinding of the teeth. The next day, hysterical symptoms, frequent efforts to vomit, convulsions, and continued agitation. In the night the patient lost all consciousness; the limbs became stiff. Dr. Meplain found his patient, on the 15th instant, in the following condition:—She was lying on her back, in a state of complete immobility; the eyelids raised, the eyes fixed, and pupils contracted; the head completely bent backwards; the jaws forcibly held together, so that they could not be opened by any efforts; the limbs in a state of tetanic rigidity; pulse scarcely perceptible; the skin cold; abdomen soft; and the urine (the last passed on the preceding day,) white and milky. Dr. Meplain, from all the previous circumstances, conceived these symptoms to be produced by the presence of worms; being taught, he says, by numerous dissections, that, in these cases, the coming on of convulsive symptoms indicates that the worms have passed into the stomach, and seek an exit by the œsophagus. He therefore attempted, by various means, which we need not detail, for the space of six hours, to bring on the action of vomiting; but without success. He then determined, considering the very critical state of the patient, to attempt the injection of an emetic into the veins. Dr. M., therefore, laid bare the median vein of the left arm, which having separated from the cellular tissue, and having passed a ligature underneath, (interposing a piece of pasteboard between it and the vein,) and drawn it out to the level of the integuments, he pushed the blood contained above the ligature upwards with his fore-finger, and then made a longitudinal incision of four or five lines in the vein, into the cavity of which he placed the canula of an ordinary trocar; this being done, he injected, by means of a hydrocele syringe, six ounces of whey, in which were dissolved four grains of tartar emetic. When this quantity was taken into the vein, the ligature was cut, the vein suffered to fall into its situation at the bottom of the wound, which was brought together and bound up, as is usual after a common bleeding.

The patient gave no sign of sensibility during the operation, which was finished at eleven minutes after two; at twenty-eight minutes after two, the eyes and the lower lip began to move. These details are continued, but at too great a length to permit us to follow them verbatim: suffice it to say, that at forty-two minutes past two vomiting took place, and eight lumbrici, rolled up in a ball, were ejected; they were all living. The amendment of the condition of the patient was striking: the rigidity of the limbs had ceased; she could swallow, but could not speak; the pulse was stronger, but intermitted; and she appeared to be fainting. At fifty-six minutes after two, she vomited again two lumbrici of a larger size; after which she began to articulate, complaining of a sense of heat in her chest, of a violent head-ache, and a sense of uneasiness, which she could not describe. From that moment the condition of the patient presented nothing remarkable. She vomited several times afterwards, bringing up, together with a prodigious quantity of bile, five more lumbrici. For three days after, the patient suffered much general disturbance of the system, head-ache, thirst, &c.; but the menses appeared on the 18th, and on the 20th she was quite recovered.—*(Jour. Comp. des Sciences Medicales*, Fevrier.)

MISCELLANEOUS.

3. *York Lent Assizes,* March 31st.—*Action to Recover the Amount of an Apothecaries' Bill.* RAEBURN v. BURN.—Mr. Brougham stated to the Jury, that this was an action brought to recover a very small sum of money, not more than 5 or £6., the amount of an apothecary's bill. The plaintiff, he observed, had been requested to attend the defendant's family, and had been some time employed in curing them of a disorder, the name of which he would not mention, but merely say that it was of that description, that the sooner they got rid of it the better, both on their own accounts, and on account of those who might have occasion to shake hands with them. Indeed, it was hard to say whether they or their friends were most interested in its speedy removal, (*a laugh.*) For that service the plaintiff had sent in a very moderate bill,—not amounting to more than 20s. for each pair of hands so cured, (*a laugh.*) The defendant had refused payment, and it therefore became necessary for the plaintiff to resort to this action.

An assistant of the plaintiff proved the regular attendance of the plaintiff, and the delivery of the medicine, ointments, liniments, &c.

—— Raeburn, brother of the plaintiff, was called to prove that the plaintiff was a regular surgeon. He stated, that the plaintiff had studied for three years in the university of Glasgow, and had been properly qualified to act as a surgeon. He had acted in that capacity from the time of his leaving Glasgow till 1814. In 1814 he had gone to sea. He had gone as a surgeon of a vessel: the name of the vessel he did not recollect, but the captain's name was Clarke.—In examination by Mr. Justice Bayley, witness could not answer, from his own knowledge, that plaintiff had acted as surgeon on-board the ship.

Mr. Justice Bayley suggested to Mr. Brougham, that this was not a case to go to the Jury. There was no proof here that the plaintiff had acted as a surgeon on the 1st of August, 1815, the date of the Act requiring all surgeons and apothecaries to undergo examination before they become qualified to practise.—Mr. Brougham submitted that there was fair ground for inferring that such had been the case. The plaintiff had been regularly educated,—had practised up to the very day of his embarkation,—and had resumed his practice immediately upon his return. He therefore thought it a fair case to go to the Jury.

Mr. Justice Bailey.—It has been already decided, that to have practised *before* the passing of the Act is not sufficient. It must be shown that the party was practising at the time when the Act passed. It is of the highest consequence that persons, practising as surgeons and apothecaries, should have a regular education.—Mr. Brougham pressed the hardship which would be felt by the plaintiff, who would be deprived by this decision of the power of recovering any of his bills.

Mr. Justice Bailey.—Why does he not get examined at the College of Surgeons or Apothecaries' Hall? I have no doubt upon the point: but, if you think this a mis-direction, you can move the Court.

The plaintiff was accordingly nonsuited.

*Fothergillian Medal.*—We have much pleasure in stating, that the Fothergillian gold medal has been conferred, by the unanimous suffrages of the Committee of Papers, on Mr. BAMPFIELD, for his Essay on Diseases of the Spine.—Our readers will remember that Mr. B. published his views on this subject in a series of valuable Papers, which appeared in this Journal last year.

### METEOROLOGICAL JOURNAL,

*From March 20, to April 19, 1824.*

**By Messrs. William Harris and Co. 50, Holborn, London.**

| | Moon. | Rain gauge | Therm. | | | Barom. | | De Luc's Hyg. | | Wind. | | Atmospheric Variation. | | |
|---|---|---|---|---|---|---|---|---|---|---|---|---|---|---|
| | | | 9 A.M. | Max. | Min. | 9 A.M. | 10 P.M. | 9 A.M. | 10 P.M. | 9 A.M. | 10 P.M. | 9 A.M. | 2 P.M. | 10 P.M. |
| Mar | | | | | | | | | | | | | | |
| 20 | | .67 | 42 | 51 | 41 | 30.13 | 30.10 | 80 | 85 | ESE | WSW | Foggy | Fair | Fair |
| 21 | | | 45 | 51 | 40 | 29.80 | 29.70 | 89 | 90 | SW | W | Overc. | Rain | Cloud. |
| 22 | ☾ | | 42 | 50 | 35 | 29.50 | 29.53 | 88 | 90 | SSW | N | Rain | Rain | Rain |
| 23 | | | 35 | 45 | 38 | 29.65 | 29.84 | 91 | 91 | N | E | Snow | Rain | Fine |
| 24 | | | 39 | 45 | 40 | 29.81 | 29.84 | 92 | 90 | ENE | NE | Rain | Fair | Fair |
| 25 | | | 42 | 44 | 35 | 29.94 | 29.95 | 85 | 80 | ENE | NE | Fair | Fine | Fine |
| 26 | | | 38 | 45 | 38 | 29.85 | 29.81 | 78 | 82 | NE | E | Fine | Chang. | |
| 27 | | | 40 | 45 | 33 | 29.70 | 29.64 | 78 | 80 | NE | N | | | Cloud. |
| 28 | | | 38 | 42 | 30 | 29.75 | 29.83 | 75 | 77 | NE | NE | | Snow | Cloud. |
| 29 | | | 37 | 45 | 37 | 29.85 | 29.76 | 74 | 74 | NNW | W | | Fine | |
| 30 | ● | | 40 | 46 | 30 | 29.60 | 29.60 | 78 | 79 | WNW | NNE | Overc. | Rain | Rain |
| 31 | | 65 / 132 | 33 | 42 | 30 | 29.56 | 29.70 | 74 | 74 | NNE | NW | Fine | Fine | Fine |
| Apr. | | | | | | | | | | | | | | |
| 1 | | | 32 | 45 | 37 | 29.77 | 29.47 | 65 | 80 | W | NW | | | Rain |
| 2 | | | 41 | 46 | 32 | 29.00 | 29.10 | 80 | 80 | W | NW | Rain | Rain | |
| 3 | | | 36 | 46 | 35 | 29.90 | 30.00 | 70 | 68 | NNE | NE | Fine | Cloud. | Cloud. |
| 4 | | | 42 | 45 | 34 | 30.13 | 30.25 | 61 | 67 | ENE | E | | Fine | Fine |
| 5 | | | 41 | 47 | 37 | 30.30 | 30.30 | 74 | 78 | NE | E | | | |
| 6 | ☽ | | 42 | 45 | 38 | 30.27 | 30.27 | 75 | 80 | NE | NE | Cloud. | Windy | Cloud. |
| 7 | | .10 | 40 | 45 | 37 | 30.03 | 30.01 | 86 | 80 | NW | ENE | Rain | Cloud. | Fair |
| 8 | | | 40 | 49 | 40 | 30.12 | 30.03 | 78 | 84 | NE | NE | Fair | Fine | Fine |
| 9 | | | 41 | 45 | 38 | 29.91 | 29.74 | 88 | 85 | NE | W | Rain | | Fair |
| 10 | | | 40 | 43 | 31 | 29.23 | 29.30 | 85 | 88 | NE | NW | | Rain | Rain |
| 11 | | | 32 | 45 | 36 | 29.40 | 29.24 | 87 | 87 | NW | WSW | Snow | Fine | Cloud. |
| 12 | | | 39 | 44 | 39 | 29.24 | 29.40 | 83 | 87 | W | WNW | Fine | | |
| 13 | ○ | | 40 | 46 | 33 | 29.52 | 29.65 | 84 | 80 | W | W | | | Fine |
| 14 | | | 41 | 49 | 37 | 29.74 | 29.74 | 70 | 74 | W | NW | | | Rain |
| 15 | | | 40 | 49 | 33 | 29.72 | 29.53 | 70 | 78 | E | E | | | Cloud |
| 16 | | | 38 | 45 | 34 | 29.15 | 29.15 | 95 | 98 | E | NNE | Rain | Rain | Rain |
| 17 | | | 40 | 45 | 34 | 29.33 | 29.60 | 98 | 85 | NE | ENE | | | Fai |
| 18 | | | 49 | 53 | 44 | 29.95 | 30.10 | 67 | 67 | ENE | ESE | Fine | Fine | Fine |
| 19 | | 120 | 50 | 54 | 44 | 30.11 | 30.15 | 65 | 70 | SSE | E | | | |

The quantity of rain fallen in the month of March, was 1 inch and 32.100ths.

